# Electrocardiographic changes mimicking acute coronary syndrome in a large intracranial tumour: A diagnostic dilemma

**DOI:** 10.1186/s12872-017-0525-2

**Published:** 2017-04-04

**Authors:** Nilukshana Yogendranathan, H.M.M.T.B. Herath, S.P. Pahalagamage, Aruna Kulatunga

**Affiliations:** National Hospital, Colombo, Sri Lanka

**Keywords:** Inferior ST segment elevated myocardial infarction, Intracranial tumour, ECG changes

## Abstract

**Background:**

ST elevation Myocardial infarction is a medical emergency. A variety of noncardiac conditions had been known to mimic the ECG changes that are seen in acute coronary syndrome. Although the common ECG changes that are documented with raised intracranial pressure are T inversions, prolongation of QT interval and sinus bradycardia, ST elevation or depression, arrhythmias and prominent U waves have also been recognized. However, ST elevations in association with primary intracranial tumours are rarely reported.

**Case presentation:**

A 68-year-old female patient with a large left sided frontoparietal sphenoidal ridge meningioma with mass effect developed sudden onset shortness of breath while awaiting surgery. Her ECG showed ST segment elevations in the inferior leads along with reciprocal T inversions in anterior leads. The patient was treated with dual antiplatelet therapy and unfractionated heparin. The ST elevations in the ECG remained static and the cardiac Troponin assay was repeatedly negative. 2D ECHO, coronary angiogram and CT pulmonary angiography were normal. The repeat noncontract CT scan of the brain revealed two small areas of haemorrhage in the tumour.

**Conclusion:**

The two mechanisms for ECG changes described in subarachnoid haemorrhage are the neurogenic stunned myocardium due to the catecholamine surge on the myocytes and stress cardiomyopathy. The same mechanisms could be the reasons for the ECG changes seen in intracranial tumours. These ECG changes could be easily misdiagnosed as acute coronary syndrome. This case emphasizes the importance of the cardiac biomarkers, 2D ECHO and coronary angiogram when confronted with such a diagnostic dilemma. Thus a more holistic analysis should be practiced in diagnosing acute coronary events in patients with intracranial pathologies to obviate a myriad of unnecessary investigations, interventions, costly treatment strategies which may well be detrimental to the patient.

## Background

Myocardial infarction (MI) is defined as a clinical or pathological event due to myocardial ischaemia causing myocardial injury or necrosis. The electrocardiogram (ECG) is the mainstay in the initial diagnosis of patients with suspected acute coronary syndrome (ACS). ST elevation myocardial infarction (STEMI) is a medical emergency, as the beneficial effects of therapy with reperfusion are greatest when performed early. But a variety of noncardiac conditions have been known to mimic the ECG changes seen in ACS. It is well recognized that certain neurological diseases present with such alterations in the ECG. The common ECG changes that are documented with raised intracranial pressure are T inversions, prolongation of QT interval and sinus bradycardia. Yet ST elevation or depression, arrhythmias, neurogenic T waves, J waves and prominent U waves have all been recognized [1–4].

However the occurrence of ST elevations in association with primary intracranial tumours is not a frequent finding in the literature. We intend to report a patient with a large meningioma presenting with ST elevations in the inferior leads on ECG with no echocardiographic (ECHO) or coronary angiographic evidence of myocardial infarction.

## Case presentation

**Fig. 1:**
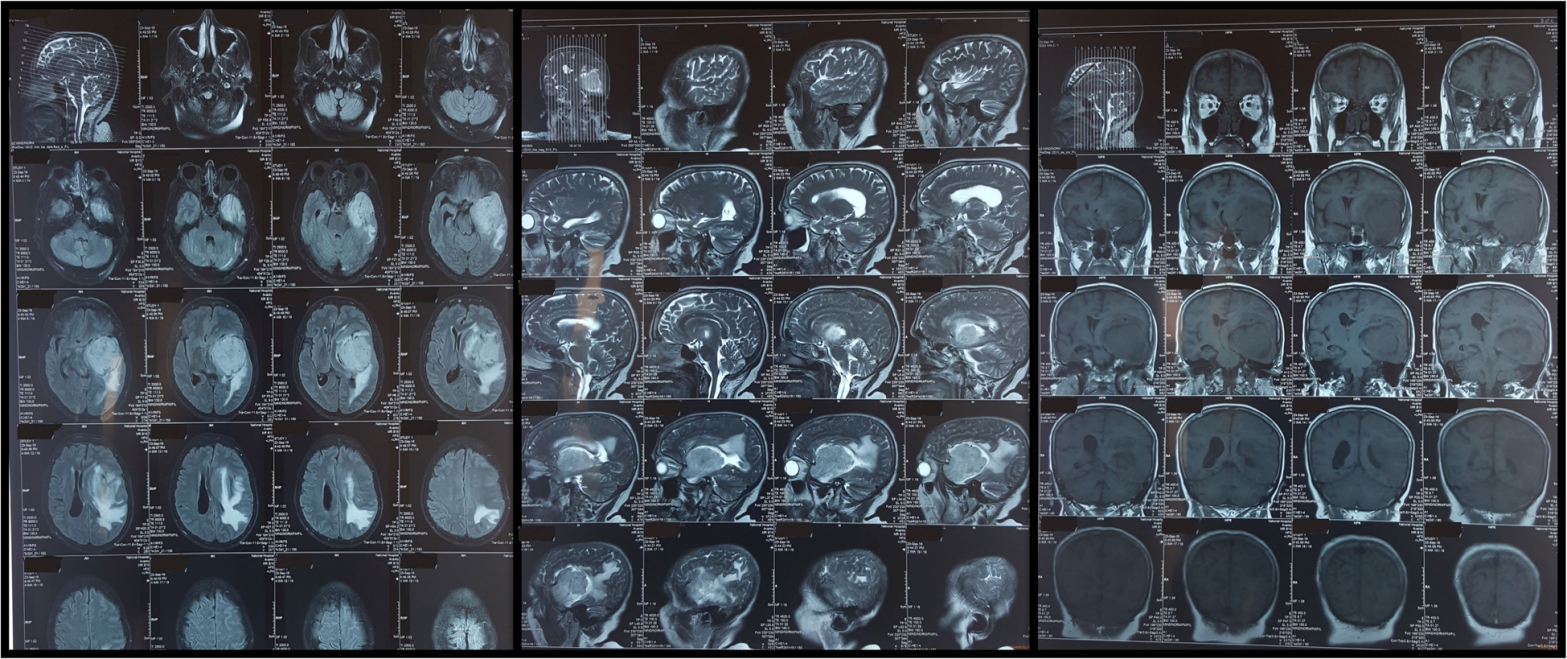
MRI images of the large left sided frontoparietal sphenoidal ridge meningioma with mass effect (Axial, sagittal and coronal view)

**Fig. 2:**
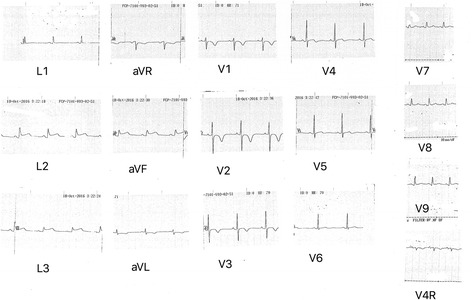
Electrocardiogram showing ST segment elevations in the inferior leads along with reciprocal T inversions in anterior leads

**Fig. 3:**
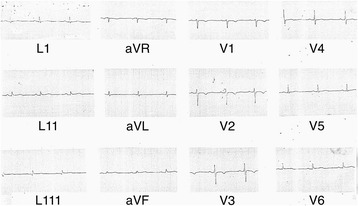
Electrocardiogram on admission

A 68-year-old female patient with the background of hypertension, hypothyroidism and dyslipidaemia was diagnosed with a large left sided frontoparietal sphenoidal ridge meningioma with mass effect (Fig. 1). She developed sudden onset shortness of breath while she was awaiting surgery in the neurosurgical ward. There was no clear history of chest pain. She was febrile and hypoxic in the room air maintaining an oxygen saturation of 90% and had bilateral diffuse crepitations in the lungs. The CXR revealed bilateral haziness of the lung fields. White cell count, C reactive protein and Erythrocyte Sedimentation Rate (ESR) were elevated (14.85 × 10^9^/ L; 30 mg/L; 85 mm at 1st hour respectively). She was commenced on intravenous broad spectrum antibiotics due to the suspicion of aspiration pneumonia. However an urgent ECG was done to exclude an acute coronary event and showed ST segment elevations in the inferior leads along with reciprocal T inversions in anterior leads (Fig. 2). Her baseline ECG on admission had been normal (Fig. 3).

**Fig. 4:**
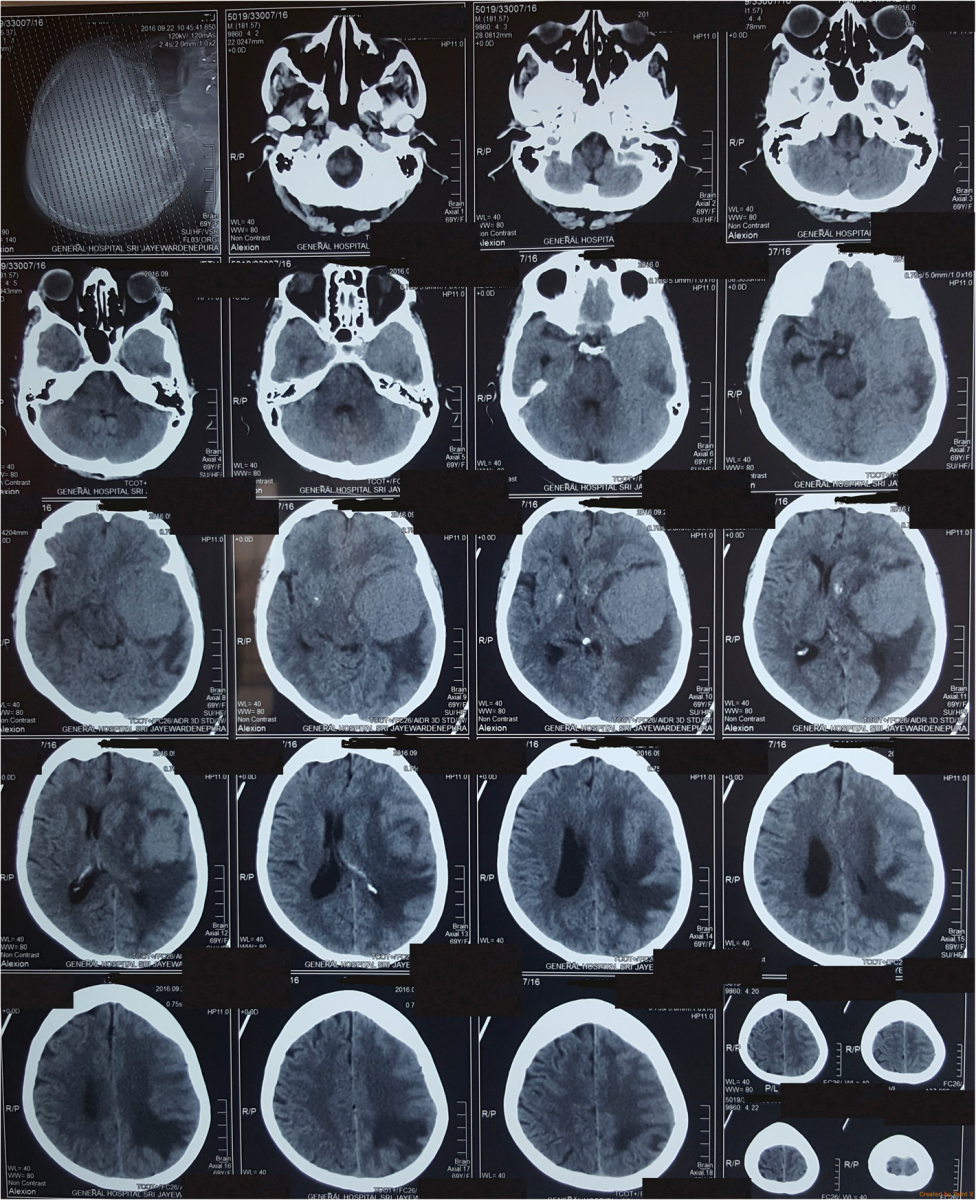
Noncontrast CT scan of the brain revealing two small areas of haemorrhage in the tumour

Then she was taken over to our acute medical unit, in liaison with the cardiology team was given loading doses of anti platelet therapy (Aspirin 300 mg and Clopidogrel 300 mg) with Atorvastatin 40 mg and then commenced on unfractionated heparin (UFH) after three hours duration from the onset of symptoms pending the results of Troponin assay. Fibrinolytic agents were not given as they are contraindicated in the presence of intracranial tumours. UFH was given as a bolus of 5000 units followed by 700 units/ h. Surprisingly the ST elevations on the ECG were static; cardiac Troponin assay done immediately and repeated three times at 4 h, 12 h and 36 h from the onset of symptoms remained negative. The Glasgow coma scale of the patient slightly deteriorated while on UFH and she was then subjected to an urgent noncontrast CT scan of the brain which revealed two small areas of haemorrhage in the tumour (Fig. 4). The UFH was discontinued by the end of 12 h and a thromboelastographic (TEG) analysis was performed with the intension to revert the coagulation abnormalities. However there was no derangement in the analysis as it was done around 12 h after the cessation of UFH. An ECHO done at 36 h from the onset of initial symptoms revealed normal sized chambers with an ejection fraction of 55%, normal aortic and mitral valves. It did not show any wall motion abnormality to suggest MI. Coronary angiography done on day 5 of the event revealed only a minor coronary artery disease with mild plaques on left circumflex artery. The D dimers too became positive with a value of 10.34 mg/L. An urgent CT pulmonary angiogram performed failed to reveal evidence of pulmonary embolism.

## Discussion

ACS is due to ischaemia of the cardiac muscles and is a medical emergency. The first step in the management is prompt recognition with proper history taking along with an ECG and cardiac biomarkers. Despite of the variety of diagnostic tests available, the ECG remains the mainstay in the initial diagnosis, categorization and the localization of the vascular territory in patients with suspected ACS. Furthermore a recent meta-analysis had suggested that presence of ST segment elevation in lead aVR as a powerful predictor of left main coronary disease and triple vessel disease [5]. Our patient had ST segment elevations confined to the inferior leads on ECG. However there had been numerous studies that concluded that there could be a myriad of ECG changes in association with raised intra cranial pressure.

A study conducted among a group of patients with elevated intracranial pressure (ICP) but without cardiac comorbidities revealed ECG changes such as ST segment elevation, T wave inversion, shortening or prolongation of QT interval, prominent U waves and notched T waves. The ICP was monitored using Konigsburg extradural transducer and the ECG changes reverted with the reduction in ICP [6].

Although the repolarisation abnormalities in ECG are more frequent after subarachnoid hemorrhages (SAH) they are also reported in brain tumours. It is believed that such changes are generally reversible and the prognostic value is limited [7]. The two mechanisms described for the ECG changes in SAH are the neurogenic stunned myocardium due to the catecholamine surge on the myocytes [8] and stress cardiomyopathy [9]. However the exact mechanisms for ST segment elevations in intracranial tumours is unclear.

There had been very few cases of intracranial tumours reported presenting with ST segment elevation. Povoa and Cavichio had concluded in a study done in Brazil that 2 out of 66 patients with intracranial tumours (3%) had ST segment elevations on ECG [10]. Hersch concluded in another study done in South Africa that 3 out of 20 (15%) patients with intracranial space occupying lesions including tumours, cerebral abscesses, subdural haematoma and tuberculoma had significant ST segment elevation in precordial leads [11].

Furthermore there had been ECG changes associated with perioperative periods of intracranial tumours [12]. Nevertheless a study conducted in Sweden had suggested that such ECG changes are more associated with SAH (15%) than intracranial tumours (13%) [13]. However, there had been recent studies concluding that the ECG changes in neurological diseases are not as frequent as reported in the literature and such changes are “non-specific” [10].

The presence of an intracranial tumour is an absolute contraindication for administration of fibrinolytic agents such as Streptokinase. Thus misdiagnosis of such ST elevations as STEMI would invariably lead to the decision to anticoagulate with short acting heparin which inturn may culminate intracranial bleeding and resulting deterioration of the neurological condition as in our patient.

## Conclusion

We present a female patient with a large intracranial tumour who presented with an ECG mimicking inferior ST elevation MI. The same mechanisms which cause ECG changes in SAH and increased ICP might be responsible for the ECG changes seen in intracranial tumours. This case also emphasises the importance of cardiac biomarkers, 2D ECHO and coronary angiogram in the diagnosis of ACS when faced with diagnostic dilemma. The ECG changes could be misleading in patients with intracranial pathologies. Thus, a more holistic analysis should be practised in diagnosing acute coronary events in such patients to obviate a myriad of unnecessary investigations, interventions, costly treatment strategies.

## Abbreviations

MI: Myocardial infarction
ECG: Electrocardiogram
ACS: Acute coronary syndrome
STEMI: ST elevation myocardial infarction
ECHO: Echocardiography
ESR: Erythrocyte sedimentation rate
UFH: Unfractionated heparin
GCS: Glasgow Coma Scale
CT: Computer tomography
TEG: Thromboelastographic analysis
ICP: Intracranial pressure
SAH: Subarachnoid hemorrhages
SDH: Subdural haemorrhages

## References

[1] Library E, Library T, 100 T, Burns E. Raised Intracranial Pressure - Life in the Fast Lane ECG Library. LITFL: Life in the Fast Lane Medical Blog. 2016 [cited 23 October 2016]. Available from: http://lifeinthefastlane.com/ecg-library/raised-intracranial-pressure/

[2] Lindberg D, Jauch E (2006). Neurogenic T Waves preceding acute ischemic stroke. Circulation.

[3] Pinto W, Barros L, Souza P, Pedroso J, Barsottini O (2014). Neurogenic T waves as clues for diagnosing hemorrhagic stroke. Arq Neuropsiquiatr.

[4] Milewska A, Guzik P, Rudzka M, Baranowski R, Jankowski R, Nowak S (2009). J-wave formation in patients with acute intracranial hypertension. J Electrocardiol.

[5] D’Ascenzo F (2012). Prevalence and non-invasive predictors of left main or three-vessel coronary disease: evidence from a collaborative international meta-analysis including 22 740 patients. Heart.

[6] Jachuck S, Ramani P, Clark F, Kalbag R (1975). Electrocardiographic abnormalities associated with raised intracranial pressure. BMJ.

[7] Clinical Disorders - ECGpedia. en.ecgpedia.org. 2016 [cited 23 Oct 2016]. Available from: http://en.ecgpedia.org/index.php?title=Clinical_Disorders

[8] Coppola G, Carità P, Corrado E, Borrelli A, Rotolo A, Guglielmo M (2013). ST segment elevations: always a marker of acute myocardial infarction?. Indian Heart J.

[9] Manikandan S (2016). Heart in the brain injured. J Neuroanaesthesiol Crit Care.

[10] Póvoa R, Cavichio L, Almeida A, Viotti D, Ferreira C, Galvão L et al. Electrocardiographic abnormalities in neurological diseases. Arq Bras Cardiol. 2003;80(4):355-6 . 10.1590/s0066-782x2003000400001 12754557

[11] Hersch C (1964). Electrocardiographic changes in subarachnoid Haemorrhage, meningitis, and intracranial space-occupying lesions. Heart.

[12] López-Lluva M, Arizón-Muñoz J, Gonzalez-Ruiz de la Herran F, Marina-Breysse M (2012). Electrocardiographic changes underlying central nervous system damage. Rev Esp Cardiol (English).

[13] Rudehill A, Olsson G, Sundqvist K, Gordon E (1987). ECG abnormalities in patients with subarachnoid haemorrhage and intracranial tumours. J Neurol Neurosurg Psychiatry.

